# Letter from the Editor in Chief

**DOI:** 10.19102/icrm.2025.16115

**Published:** 2025-11-15

**Authors:** Devi Nair


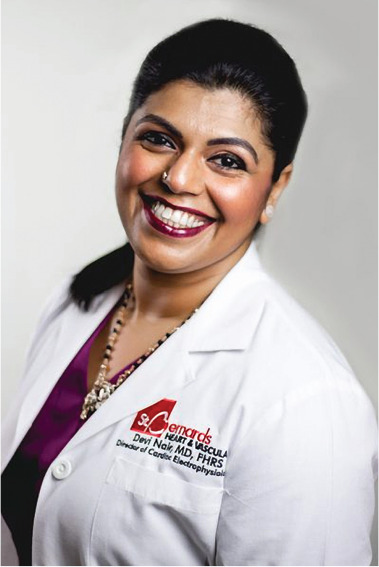


Dear Colleagues,

November was a remarkable month for our global electrophysiology community, as two major international meetings—APHRS in Yokohama and LAHRS in Buenos Aires—brought together clinicians, scientists, and educators from every region of the world. These meetings underscored the increasingly interconnected nature of rhythm science and highlighted how advances in imaging, device therapy, ablation, and multidisciplinary care transcend geographic boundaries.

## A Global Perspective: APHRS and LAHRS

At APHRS this year, the focus remained on innovation in atrial fibrillation management, pulsed field ablation, physiologic pacing, and digital integration. Sessions highlighted the rapid adoption of new technologies across Asia and emphasized the importance of scalable training pathways as electrophysiology capabilities continue to expand internationally.

Meanwhile, LAHRS showcased the power of regional collaboration in Latin America, with a strong emphasis on cardiac imaging in electrophysiology, novel implantable cardioverter-defibrillator (ICD) technologies, and nursing- and patient-centered approaches to device therapy. The meeting also reinforced the critical role of equitable access—ensuring that advancements in rhythm management translate into real-world improvements across diverse health systems.

These two global gatherings—held on opposite sides of the world—shared a unifying theme: progress accelerates when we learn together.

This issue of the journal reflects that same spirit of collaboration and innovation.

## Scientific Highlights from This Issue

Dragisic et al. report encouraging early experience with the Aurora EV-ICD™ (Medtronic, Minneapolis, MN, USA) in adolescents with genetic arrhythmias and hypertrophic cardiomyopathy.^1^ All implantations were successful, with stable sensing and no complications throughout follow-up. Their work demonstrates the feasibility and safety of extravascular ICD therapy in a population traditionally underserved by conventional ICD technology.

Polikandrioti et al. explore the essential role of specialized nursing care in optimizing outcomes for patients with cardiac implantable electronic devices.^2^ Structured education, emotional support, and patient engagement were shown to significantly improve quality of life, reduce anxiety, and strengthen adherence—underscoring that successful device therapy relies not only on advanced technology but also on comprehensive, human-centered care.

Raharjo and Zharfan provide an extensive, forward-looking review of cardiac imaging across the spectrum of electrophysiology—from diagnosing arrhythmogenic cardiomyopathy to guiding atrial and ventricular ablation, device implantation, and conduction system pacing.^3^ Their discussion extends into the emerging frontier of digital twin modeling, where multimodal imaging and electrophysiologic data converge to simulate cardiac behavior and enhance procedural planning. This review captures the growing role of imaging as the backbone of precision electrophysiology.

Finally, Schaller and El-Chami offer a timely review of how transcatheter tricuspid valve replacement (TTVR) is reshaping cardiac device planning.^4^ Because transvenous leads crossing the tricuspid valve can become entrapped or dysfunctional after TTVR, the authors outline modern alternatives to avoid this problem—including leadless pacing, coronary sinus–based systems, subcutaneous and extravascular ICDs, and surgical epicardial approaches. They emphasize the need for proactive, multidisciplinary planning to avoid complications such as lead jailing, loss of extractability, and pacing-induced tricuspid regurgitation. As TTVR expands rapidly, device strategies must evolve in parallel to ensure long-term safety and adaptability.

## Unifying Themes Across Continents

What unites the scientific work in this issue with the global insights from APHRS and LAHRS is a common purpose: to refine our tools, strengthen our understanding, and improve the patient experience across every stage of rhythm care.

Whether through imaging-enhanced precision, innovative device platforms, or patient-centered models of care, the field continues to evolve with remarkable speed—and remarkable cooperation.

## Looking Ahead

As we approach the end of 2025, our international community stands more connected than ever before. 

The November meetings in Yokohama and Buenos Aires demonstrated that the challenges we face—and the opportunities we embrace—are shared across continents.

We remain proud to serve as a platform where these ideas, experiences, and innovations converge, and we look forward to continuing this global dialogue in the months ahead.

Warm regards,


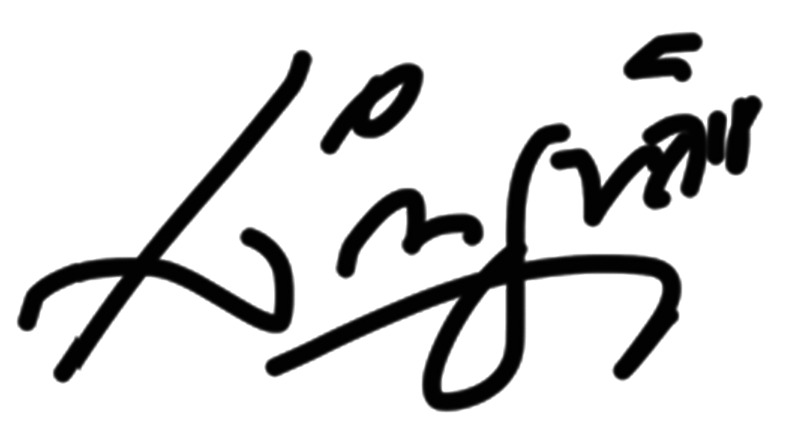


Dr. Devi Nair, md, facc, fhrs

Editor-in-Chief

*The Journal of Innovations in Cardiac Rhythm Management*

Director of the Cardiac Electrophysiology & Research,

St. Bernard’s Heart & Vascular Center, Jonesboro, AR, USA

White River Medical Center, Batesville, AR, USA

President/CEO, Arrhythmia Research Group

Clinical Adjunct Professor, University of Arkansas for Medical Sciences

Governor, Arkansas Chapter of the American College of Cardiology

drdgnair@gmail.com
